# Early endoscopic treatment of symptomatic pancreatic necrotic collections

**DOI:** 10.1038/s41598-021-03924-2

**Published:** 2022-01-10

**Authors:** Mateusz Jagielski, Jacek Piątkowski, Marek Jackowski

**Affiliations:** Department of General, Gastroenterological and Oncological Surgery, Collegium Medicum Nicolaus Copernicus University, 53-59 Św. Józefa St, 87-100 Toruń, Poland

**Keywords:** Diseases, Gastroenterology, Medical research

## Abstract

EUS-guided transmural endoscopic drainage is commonly used in the treatment of WOPN in the late phase of ANP. The role of endoscopic intervention remains unclear in the early phase of ANP. This study aimed to prospectively evaluate early endoscopic treatment of ANCs compared with endoscopic drainage of WOPN. Overall, 71 patients with ANP who underwent transmural endoscopic drainage for necrotic collections were included. Endoscopic intervention was performed within the first four weeks of ANP in 25 (35.21%) patients with ANC (Group 1) and in 46 (64.79%) patients after four weeks since the onset of ANP with WOPN (Group 2). The overall mean age of patients was 49.9 (22–79) years and 59 of them were males. The mean time of active drainage and duration of total endoscopic treatment was 26.8 and 16.9 days (*P* = 0.0001) and 270.8 and 164.2 days (*P* = 0.0001) in Groups 1 and 2, respectively. The average total number of endoscopic interventions was 9.5 and 4.5 in Groups 1 and 2, respectively (*P* = 0.0001). The clinical success rate, frequency of complications of endoscopic interventions, long-term success rate, and recurrence rate were not significantly different between the groups (*P* > 0.05 for each). Transmural endoscopic drainage is effective method of treatment of early ANCs within the first four weeks of ANP. However, compared with endoscopic intervention in WOPN, more interventions and longer duration of drainage are required.

## Introduction

Acute necrotizing pancreatitis (ANP) is seen in approximately 20%–40% of patients with acute pancreatitis^1^. It can lead to necrosis of the pancreatic parenchyma (central necrosis), peripancreatic tissues (peripheral necrosis), or both (mixed necrosis)^2–4^. Abnormal lesions of the pancreas or peripancreatic tissues on imaging within 4 weeks of ANP are called acute necrotic collections (ANCs). Beyond 4 weeks, they may evolve to walled-off pancreatic necrosis (WOPN), which contains liquefied necrosis and fragments of necrotic tissues^2–5^. Over time, the number of solid fragments lessens within the liquefied contents and the wall of the collection becomes thicker^2–5^. ANCs are observed in most patients with ANP^4,5^. Almost half of all ANCs regresses spontaneously, while the other half tends to evolve into WOPN^4,5^. In half of the patients with WOPN, the fluid collection undergoes spontaneous resorption without a need for intervention^4–6^. The other half of the patients develops symptoms related to fluid collection, which is an indication for interventional treatment^4–6^.

The management of patients with ANP depends mainly on the patient’s clinical condition both in early and late phase of disease^7–9^. In most cases the optimal therapeutic strategy in ANP is intensive conservative management in the early phase followed by interventional treatment (drainage with or without necrosectomy) in the late phase of the disease^7–9^.

If patient’s condition does not improve despite intensive conservative treatment in the early phase of ANP and intervention is still necessary, then minimally invasive procedures (step up approach) should be preferred^7–11^, such as percutaneous transperitoneal or retroperitoneal drainage^10^. Only when minimally invasive procedures are ineffective, laparotomy with debridement (open necrosectomy) remains the treatment of choice, which is characterized by high morbidity and mortality rates^12^. Therefore, open necrosectomy is reserved for patients with ANP who do not improve despite undergoing optimal conservative treatment and minimally invasive procedures^1,12^.

Interventional treatment for pancreatic necrosis should be postponed by at least four weeks since the onset of ANP^7,8,13^. That is when the necrotic contents becomes liquified and necrotic collection becomes walled-off^7,8,13^. Delaying intervention in ANP reportedly improves the therapeutic outcomes with reduced morbidity and mortality rates^7,10,11^; however, these studies were only concerned with the surgical interventions^14^. Data on early endoscopic intervention in managing the sequelae of ANP are lacking. The use of minimally invasive techniques in managing ANP sequelae significantly reduces the complication rate compared with open necrosectomy^12^. Transmural endoscopic drainage, as a minimally invasive procedure, is a widespread method of managing WOPN in the late phase of acute pancreatitis^15^. The role of endoscopic treatment in early phase of ANP remains unclear when interventional treatment for ANCs is necessary.

This study aimed to prospectively evaluate endoscopic drainage in early ANC compared with that in WOPN in late phase of ANP.

## Methods

The study was approved by the Ethics Committee of Institutional Review Board (Collegium Medicum Nicolaus Copernicus University) and proceeded in line with the tenets set by the Declaration of Helsinki.

In 2019, 184 patients were treated in our medical center for acute pancreatitis and its consequences. Of them, 93 (50.54%) patients were diagnosed with ANP. 132/184 (71.74%) patients had previously been treated for acute pancreatitis in other hospitals and later transferred to our reference center for the treatment of consequences—mainly pancreatic and peripancreatic fluid collections. The diagnosis of acute pancreatitis, the clinical and morphological classification criteria, and all definitions of local and systemic complications were based on the 2012 revision of the Atlanta classification^2,3^. All patients with acute pancreatitis were assessed using APACHE II (Acute Physiology And Chronic Health Evaluation II), SOFA (Sequential Organ Failure Assessment), and Ranson scores at the time of admission (day 0) and on day 2 of hospitalization^2,3,16–18^.

The standard for management of acute pancreatitis in present study is not different from the international guidelines^16,17^. The mainstay of treatment remained nutritional support with intensive intravenous fluid replacement and analgesics, depending on the associated organ dysfunction and the clinical state.

Abdominal contrast-enhanced computed tomography (CECT) was conducted in every patient with severe acute pancreatitis when infected necrosis was suspected based on no clinical improvement within the first 48 h of hospitalization despite treatment. CECT findings were evaluated using the Computed Tomography Severity Index (CTSI) score^2,3,18^.

Both medical records and imaging studies in each case of acute pancreatitis were reviewed during interdisciplinary meetings of senior staff in our medical center and the final decisions regarding further management and need for interventional treatment were made.

### Inclusion criteria

All patients with symptoms related to necrotic collections (ANC or WOPN) who underwent endoscopic drainage based on the clinical presentation and imaging studies were enrolled. Endoscopic intervention was delayed till the fluid collection developed a wall and the necrotic contents liquefied into WOPN, which usually takes 4 weeks since the onset of the disease, as confirmed on abdominal imaging. If a patient’s condition did not improve despite intensive optimal conservative treatment in the early phase of ANP, endoscopic intervention was performed within 4 weeks of acute pancreatitis (Group 1). In the remaining patients, endoscopic treatment was performed after 4 weeks since the onset of the disease (Group 2). The patients’ characteristics are presented in Table 1.

**Table 1:** Detailed characteristics of patients of the two groups.

	ANC (*N* = 25)	WOPN (*N* = 46)	Total (*N* = 71)	*P*-value
**Sex**				0.1401
Female	2 (8.0%)	10 (21.7%)	12 (16.9%)	
Male	23 (92.0%)	36 (78.3%)	59 (83.1%)	
**Age (years)**				0.0114
Mean (SD)	44.0 (14.4)	53.2 (13.9)	49.9 (14.7)	
Range	22.0–74.0	25.0–79.0	22.0–79.0	
Median	42.0	56.0	50.0	
95% CI	38.1–50.0	49.0–57.3	46.5–53.4	
**Etiology**				0.0699
Alcoholic	20 (80.0%)	27 (58.7%)	47 (66.2%)	
Non-alcoholic	5 (20.0%)	19 (41.3%)	24 (33.8%)	
**Time between onset of pancreatitis and intervention (days)**				0.0001
Average (SD)	16.4 (4.9)	74.5 (45.9)	54.0 (46.3)	
Range	8.0–25.0	30.0–240.0	8.0–240.0	
Median	16.0	56.5	44.0	
95% CI	14.3–18.4	60.9–88.1	43.1–65.0	
**Ranson score (day 0)**				0.1662
Mean (SD)	1.7 (1.1)	1.3 (1.0)	1.5 (1.1)	
Range	0.0–4.0	0.0–4.0	0.0–4.0	
Median	2.0	1.0	1.0	
95% CI	1.3–2.2]	1.0–1.6	1.2–1.7	
**Ranson score (day 2)**				0.0186
Mean (SD)	3.3 (1.3)	2.5 (1.2)	2.8 (1.3)	
Range	1.0–6.0	0.0–5.0	0.0–6.0	
Median	3.0	2.0	3.0	
95% CI	2.8–3.9	2.2–2.9	2.5–3.1	
**APACHE II score**				0.1324
Mean (SD)	12.7 (4.1)	11.0 (4.3)	11.6 (4.3)	
Range	6.0–20.0	2.0–21.0	2.0–21.0	
Median	13.0	10.5	11.0	
95% CI	11.0–14.4	9.7–12.2	10.5–12.6	
**SOFA score**				0.0143
Mean (SD)	3.8 (2.2)	2.5 (1.1)	3.0 (1.7)	
Range	1.0–8.0	0.0–5.0	0.0–8.0	
Median	4.0	2.5	3.0	
95% CI	2.9–4.7	2.1–2.8	2.6–3.4	
**CTSI**				0.2062
Mean (SD)	8.1 (1.3)	7.7 (1.3)	7.8 (1.3)	
Range	6.0–10.0	5.0–10.0	5.0–10.0	
Median	8.0	7.0	8.0	
95% CI	7.6–8.7	7.3–8.0	7.5–8.1	
**Initial size of necrotic collection (mm)**				0.0001
Mean (SD)	185.2 (68.1)	123.0 (47.7)	144.9 (62.8)	
Range	88.0–320.0	68.0–247.0	68.0–320.0	
Median	178.0	117.0	130.0	
95% CI	157.1–213.3	108.9–137.2	130.1–159.8	
**Percentage of necrosis**				0.0027
25%–50%	0 (0.0%)	9 (19.6%)	9 (12.7%)	
50%–75%	7 (28.0%)	22 (47.8%)	29 (40.8%)	
> 75%	18 (72.0%)	15 (32.6%)	33 (46.5%)	
**Type of necrosis**				0.0132
Central	0 (0.0%)	9 (19.6%)	9 (12.7%)	
Peripheral	0 (0.0%)	4 (8.7%)	4 (5.6%)	
Mixed	25 (100.0%)	33 (71.7%)	58 (81.7%)	

SD, standard deviation; CI, confidence interval; ANC, acute necrotic collection; WOPN, walled-off pancreatic necrosis.

The main two indication group to begin endoscopic treatment of necrotic collections are: symptoms related to mass effect of the collection, and infection of the necrotic content.

The symptoms related to the mass effect as an indication for interventional treatment were defined as pressure on neighboring organs caused by the necrotic collection. Mechanical icterus specified as clinical and radiological symptoms of mechanical obstruction of extrahepatic bile ducts, was a result of impression of the collection on the common bile duct. Subiles or ileus were defined as clinical and radiological signs of gastrointestinal tract obstruction by the necrotic collections. Abdominal compartment syndrome (ACS) was defined as new organ dysfunction or failure related to the intra-abdominal pressure above 20 mmHg. In the late phase of ANP abdominal pain and weight loss as indications for endoscopic treatment were also related to the mass effect of the collection. Abdominal pain was never a single indication for endotherapy. It was always accompanied by other symptoms listed in Table 2.

**Table 2:** Indications for endoscopic treatment.

	ANC (*N* = 25)	WOPN (*N* = 46)	Total (*N* = 71)	*P*-value
**Indication**				
Infection	16 (64.0%)	20 (43.5%)	36 (50.7%)	0.0985
Subileus/ileus	8 (32.0%)	15 (32.6%)	23 (32.4%)	0.9583
Icterus	0 (0.0%)	5 (10.9%)	5 (7.0%)	0.0873
Abdominal pain	0 (0.0%)	15 (32.6%)	15 (21.1%)	0.0013
Weight loss	0 (0.0%)	12 (26.1%)	12 (16.9%)	0.0051
Abdominal compartment syndrome	6 (24.0%)	0 (0.0%)	6 (8.5%)	0.0005

The infection of necrotic collection as an indication for interventional treatment was defined as lack of clinical improvement or deterioration of patient’s clinical status despite use of broad spectrum antibiotic therapy in patients with confirmed infection of the necrotic collection. That group of indications includes septic shock related to collection’s infection.

To summarize inclusion criteria and strategy of management of patients with pancreatic necrosis—in this study interventional treatment for symptomatic pancreatic necrosis was postponed by at least four weeks since the onset of ANP, when the necrotic contents liquified and necrotic collection became walled-off. However, in some cases postponing of the interventional treatment was not possible, because patient’s condition did not improve despite maximal intensive conservative treatment. In these cases interventional treatment within first four weeks since the onset of ANP was necessary. The inclusion criteria for interventional treatment in an early phase of ANP were: infection of the ANC with accompanying sepsis or ileus caused by mass effect of the ANC or ACS. These were only three criteria for interventional treatment of ANC in this study.

### Exclusion criteria

Patients with pancreatic fluid collections associated with acute pancreatitis other than ANC or WOPN were excluded. Additionally, patients with asymptomatic ANC or WOPN were exempted. Patients with previous diagnosis of chronic pancreatitis were also excluded. Patients who had previously undergone pancreatic surgery were exempted. Patients who underwent surgeries except endoscopic intervention for pancreatic necrosis were excluded.

### Algorithm for the type of endoscopic treatment

In symptomatic patients with necrotic collections, transmural drainage was performed when the distance between the collection wall and gastrointestinal tract was < 30 mm on endoscopic ultrasound (EUS). Direct endoscopic necrosectomy (DEN)^15^ was performed when there was no clinical improvement despite drainage or infected necrotic collection. If single transluminal gateway techniques (SGT)^15,19^ proved unsuccessful and necrosis spread outside the lesser sac, single transluminal gateway transcystic multiple drainage (SGTMD)^20–22^ was performed to access extensive areas of necrosis from a single transluminal gateway. If there were multiple non-communicating necrotic collections, multiple transluminal gateway techniques (MTGT)^22,23^ were used.

### Endoscopic procedures

Endoscopic procedures were performed under general anesthesia with endotracheal intubation. All patients provided informed consent for such treatment. Endoscopic procedures were performed using a linear echoendoscope (Pentax EG3870UTK), duodenoscope (Olympus TJF-Q180V), and gastroscope (Olympus GIF-H185) with insufflation with carbon dioxide. In all patients, endoscopy was performed by the same operator. All patients received antibiotic prophylaxis (ciprofloxacin or ceftriaxone) beforehand. Samples of the contents were sent for cultures, cytology, and laboratory tests.

#### Single transluminal gateway techniques (SGT)^15,19,22^

Fistulotomy was located with use of EUS. Enterostomy was performed using cystotome (Cystotome CST-10, Cook Endoscopy). Fistula between the gastrointestinal tract lumen and necrotic collection was widened using a 15-mm high-pressure balloon (Cook Endoscopy or Boston Scientific). Through the fistula, a lumen-apposing metal stent (LAMS) (diameter, 16 mm; length, 30 or 40 mm Taewoong Medical or Olympus) was inserted transmurally in each case. Subsequently, through the LAMS, 7- or 8-Fr nasal drain (Cook Endoscopy) and/or a 7- or 8.5-Fr double pigtail endoscopic stent (Cook Endoscopy) were inserted.

#### Multiple transluminal gateway techniques (MTGT)^22,23^

In patients qualified for creation of another transmural tract between the necrotic cavity and the gastrointestinal tract, the site of fistulotomy was also determined under EUS guidance. Enterostomy was performed using cystotome (Cystotome CST-10, Cook Endoscopy). The fistula was dilated using a high-pressure 15-mm balloon (Cook Endoscopy or Boston Scientific). Subsequently, a LAMS (diameter, 16 mm; length, 30 or 40 mm Taewoong Medical or Olympus) was inserted transmurally. Through the LAMS, a 7- or 8-Fr nasocystic drain (Cook Endoscopy) and/or a 7- or 8.5-Fr double pigtail endoscopic stent (Cook Endoscopy) were inserted into the necrotic collection.

#### Single transluminal gateway transcystic multiple drainage (SGTMD)^20–22^

In patients qualified for SGTMD subsequent endoscopic procedures were performed and a guidewire was introduced in the necrotic subcavities with fluoroscopy guidance through the transmural tract created between the necrotic collection and the gastrointestinal lumen. The canals between the necrotic subcavities were dilated with a 8-mm high-pressure balloon (Boston Scientific) under fluoroscopy and endoscopy guidance. Subsequently, another 7- or 8-Fr nasal drain (Cook Endoscopy) and/or 7- or 8.5-Fr double pigtail stent (Cook Endoscopy) were introduced through those canals and their distal ends were deployed within necrotic subcavities.

#### Direct endoscopic necrosectomy (DEN)^15^

The nasal drain was removed and the gastroscope was inserted into the necrotic collection through the transmural stent. The collection was flushed several times with saline and the necrotic content was removed. Using a 15–20-mm extraction balloon (Cook Endoscopy) and Dormia basket (Cook Endoscopy or Olympus), the necrotic tissues were removed under direct endoscopic guidance. The procedure was repeated multiple times during each necrosectomy session. Subsequently, a nasal drain and/or double pigtail plastic stent were inserted transmurally into the necrotic collection.

### Drainage system^22,24,25^

Necrotic collection was flushed with saline (60–200 mL) through the nasal drain every 2 h for the first 48 h and every 4–6 h over the next days of active drainage. When there was clinical suspicion of ANC/WOPN infection the use of antibiotics was prolonged and another microbial culture with antibiogram of necrotic collection contents was performed.

### Monitoring^22,24,25^

The size of the necrotic collection was verified every 7 days using abdominal ultrasound. Abdominal CECT was performed to verify remission or when a patient’s condition deteriorated despite treatment. Duration of active drainage was defined as period from introduction of nasal drain to its removal. Active drainage was discontinued after clinical success was achieved.

### Definitions^22,24,25^

Complications were divided into early complications (occurring up to 30 days after treatment) and late complications (occurring more than 30 days after treatment).

Clinical success was defined as the lack of collection-related symptoms and total regression of the collection or collection diameter < 40 mm on imaging.

Long-term success was defined as the lack of symptoms and total regression of the collection or collection diameter < 40 mm after 1 year of follow-up since discontinuation of active drainage.

Recurrence of the collection was determined as the collection size > 40 mm or relapse of symptoms during a follow-up.

Duration of endotherapy was defined as the total time of endoscopic treatment (period of passive transmural drainage- from removal of nasal drain to removal of transmural stents).

### Statistical analysis

Statistical analyses were performed using StatSoft statistical package Inc. data analysis software system version 12.0 (2014, STATISTICA, Tulsa, Oklahoma, USA). Quantitative data are presented as mean, standard deviation, median range, and 95% confidence intervals (95% CIs). Qualitative data are presented as number and percentage. Shapiro–Wilk test was used to test normality of distributions. The hypothesis of the same variances was verified using the Levene's test (Brown–Forsythe test). The significance of differences between two groups (independent variables) was tested using Student’s *t*-test (or Welch test for non-homogeneous variance) or Mann–Whitney’s U test, as appropriate. Chi-squared test was used for qualitative variables (with Yates correction for < 10 cells, Cochran’s assumptions, and Fisher's test). The strength and direction of correlation between variables was evaluated using correlation analysis and Pearson’s and/or Spearman’s rank correlation coefficient. Statistical significance was defined as *P* < 0.05.

## Results

### Patient characteristics

This study included all consecutive 71 patients (59 males; mean age, 49.9 [range, 22–79] years) with ANP who underwent transmural endoscopic drainage for necrotic collections. The patients’ characteristics together with detailed information about necrotic collections are presented in Table 1.

**Figure 1:**
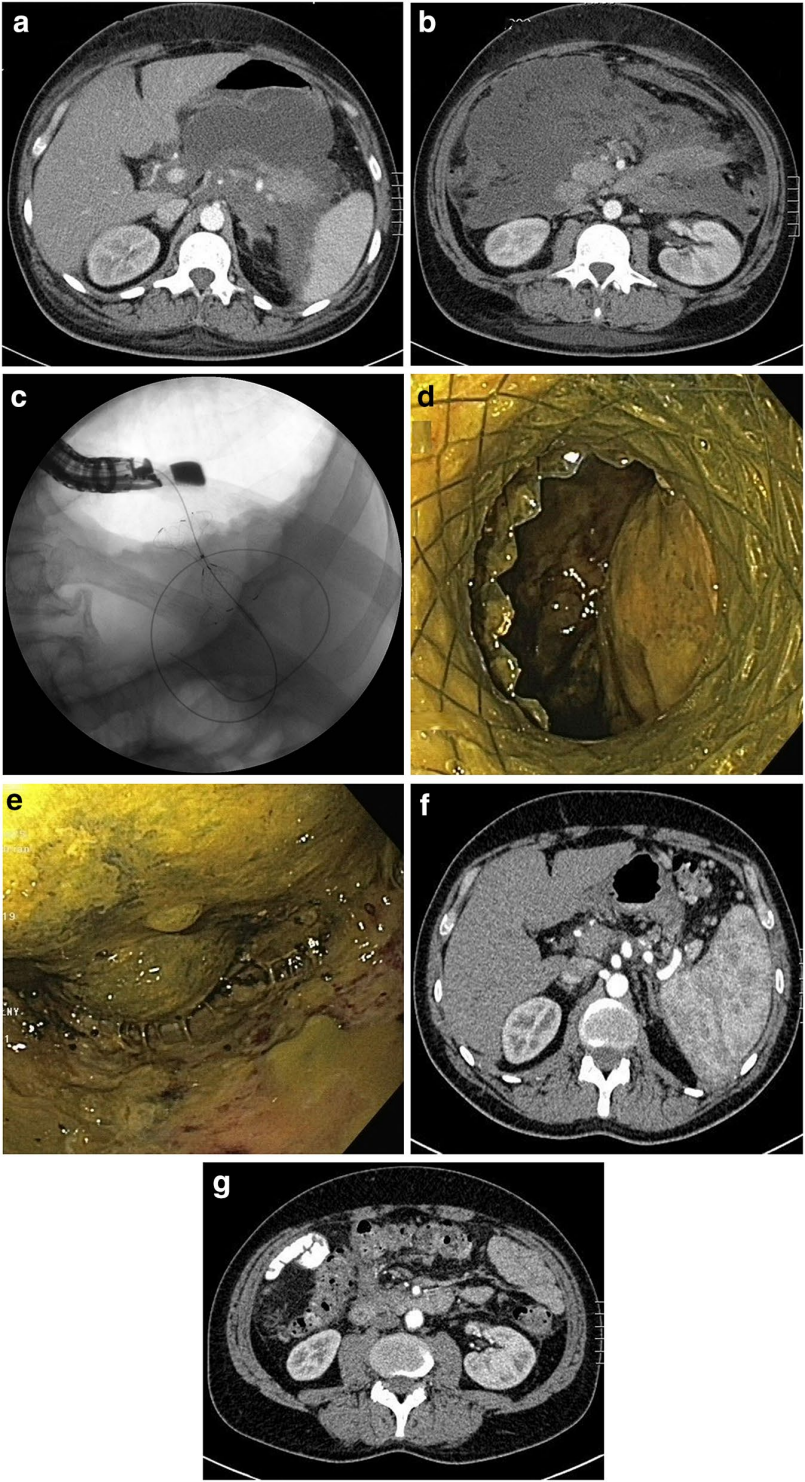
**a**–**g** Endoscopic treatment of acute necrotic collections. A 35-year-old male with acute necrotizing pancreatitis. On contrast-enhanced abdominal and pelvic computed tomography on day 8 of pancreatitis, extensive acute necrotic collections are visible (**a**, **b**). Because of symptoms of abdominal compartment syndrome, transmural endoscopic drainage of acute necrotic collections was performed. On fluoroscopy, the drainage system can be seen (**c**). During direct endoscopic debridement, solid necrotic masses within the lumen are visible (**d**, **e**). On control multiphase contrast-enhanced abdominal and pelvic computed tomography (**f**, **g**) after 6 months since the endoscopic treatment, complete regresion of necrotic collections was noted.

**Figure 2:**
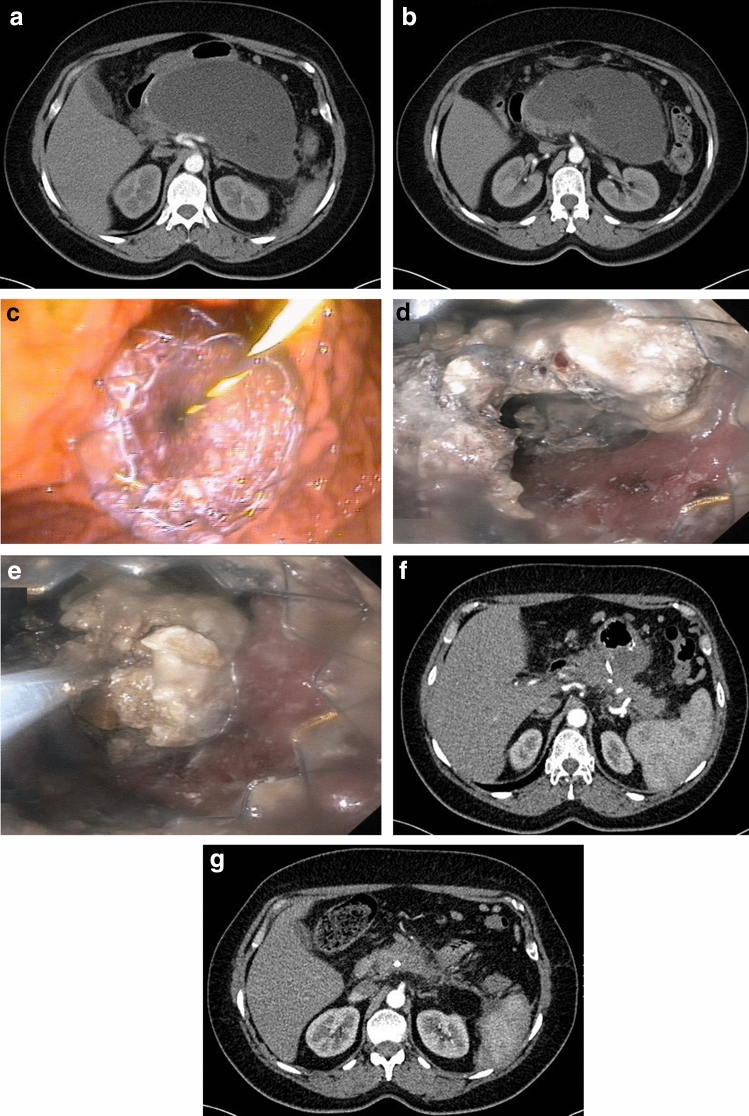
**a**–**g** Endoscopic treatment of walled-off pancreatic necrosis. A 62-year-old female patient qualified for drainage of infected walled-off pancreatic necrosis on day 48 of acute necrotizing pancreatitis (**a**, **b**). Transmural endoscopic drainage (**c**) and numerous direct endoscopic necrosectomy procedures were performed (**d**, **e**). On control contrast-enhanced abdominal and pelvic computed tomography (**f**, **g**) after 12 months since the endoscopic treatment, a complete regression of necrotic collections was noted.

Group 1 (ANC) included 25 (35.21%) patients with average time between onset of the disease and endoscopic treatment of 16.4 (8–25) days (Fig. 1a-g). Group 2 (WOPN) included 46 (64.79%) patients with average time between disease’s onset and endoscopic treatment of 74.5 (30–240) days (Fig. 2a-g). Infection of necrotic collection was an indication for endoscopic treatment in 16 (64%) and 20 (43.5%) in Groups 1 and 2, respectively (*P* = 0.0985). In both groups, the most common pathogens isolated from necrosis included *Escherichia coli, Klebsiella pneumoniae, Enterococcus faecalis,* and *Staphylococcus epidermidis.* Other indications for endoscopic therapy are presented in Table 2. In 21 (45.65%) patients in Group 2, there were multiple indication for endoscopic treatment.

Detailed results of blood tests of study group are presented in Table 3.

**Table 3:** Parameters in laboratory blood test on day of the beginning of the endoscopic treatment.

Parameter in blood test	ANC (*N* = 25)	WOPN (*N* = 46)	*p*-value
Hemoglobin, *g/dl,* mean, (SD) [range]	12.6 (3.1) [8.3–18.4]	13.8 (2.80) [8.6–17.7]	0.144
Leukocytes, *mm*^*3*^, mean, (SD) [range]	18.5 (6.8) [7.7–32.08]	13.09 (7.2) [6.1–31.01]	0.008
Thrombocytes, *mm*^*3*^, mean, (SD) [range]	489.1 (133.8) [154.0–553.0]	292.9 (125.9) [110.0–555.0]	0.013
C-reactive protein, *mg/L*, mean, (SD) [range]	225.7 (110.6) [58.8- 444.2]	252.8 (105) [49.9–504.6]	0.200
Procalcitonin, *µg/L*, mean, (SD) [range]	3.65 (5.2) [0.09- 23.4]	2.21 (3.5) [0.05–13.5]	0.185
Creatinine, *mg/dl*, mean, (SD) [range]	2.0 (0.9) [0.8–2.6]	1.8 (0.8) [0.8–2.8]	0.052
Amylase, U/L	139.8 (119.4) [30–590]	109.7 (76.8) [23–334]	0.230
Lipase, U/L	117.9 (41.4) [51–222]	82.5 (35.7) [23–166]	0.015
Bilirubin, *mg/dl*, mean, (SD) [range]	2.3 (3.0) [0.4–13.6]	1.9 (2.3) [0.5–10.0]	0.204
AST, *U/L*, mean, (SD) [range]	226.7 (230.6) [45–1105]	230 (184.4) [34–652]	0.812
ALT, *U/L*, mean, (SD) [range]	218.9 (213.4) [51–1015]	253.9 (205.2) [34–782]	0.623
D-dimer, mg/L	1.28 (0.9) [0.39–4.6]	0.96 (0.7) [0.5–3.55]	0.731

### Endoscopic treatment technique

All patients underwent transmural endoscopic drainage of necrotic collections (transgastric, *n* = 70; transduodenal, *n* = 1). No other minimally invasive techniques of treatment of pancreatic necrosis except endotherapy was used in any of treated patients. In Groups 1 and 2, SGT were used in 40% and 67.4% of patients, respectively; MTGT were used in 36% and 17.4% of patients, respectively; and SGTMD was used in 24% vs 15.2% of patients, respectively (*P* = 0.0770). DEN was performed in 21 (86%) patients in Group 1 and 14 (30.43%) patients in Group 2 (*P* = 0.0001).

### Therapy duration and efficacy

The average duration of active drainage was 26.8 (15–56) days in Group 1 and 16.9 (6–47) days in Group 2 (*P* = 0.0001). The average number of endoscopic interventions per patient was 9.5 (4–15) in Group 1 and 4.5 (2–10) in Group 2 (*P* = 0.0001). The average duration of endoscopic treatment was 270.8 (146–383) days in Group 1 and 164.2 (28–412) days in Group 2 (*P* = 0.0001). Clinical success was achieved in 23 (92%) patients in Group 1 and 44 (95.7%) patients in Group 2 (*P* = 0.5238).

### Early complications

Complications resulting from the endoscopic treatment were observed in 7 (28%) patients in Group 1 and in 11 (23.9%) patients in Group 2 (*P* = 0.7054) (Table 4). None of the patients required surgical intervention due to complications of endoscopic treatment.

**Table 4:** Complications of endoscopic treatment.

	ANC (*N* = 25)	WOPN (*N* = 46)	Total (*N* = 71)
**Complication (method of complication’s treatment)**			
Gastrointestinal bleeding (required transfusions)	4 (16.0%)	6 (13.04%)	10 (14.08%)
Transmural stent migration into lumen of the collection (treated endoscopically)	3 (12.0%)	4 (8.7%)	7 (9.86%)
Gastrointestinal perforation (treated conservatively)	0 (0.0%)	1 (2.17%)	1 (1.4%)

### Mortality

The mortality rate was 4% (1/25) in Group 1 and 4.3% (2/46) in Group 2 (*P* = 0.9445). In all cases, death was caused by multiorgan failure resulting from ANP.

### Long-term success

The average follow-up duration was 14 (10–20) months. One of the patients committed suicide during the follow-up in the 10^th^ month of observation.

Long-term success was observed in 21 (84%) patients in Group 1 and 36 (84.8%) patients in Group 2 (*P* = 0.9306). Recurrence of the collection was observed in 3 (12%) patients in Group 1 and 6 (13%) patients in Group 2 (*P* = 0.9000). Endoscopic drainage was performed in all patients with recurrence of the collection.

### Late complications

Newly diagnosed insulin-dependent diabetes was observed in 7 (28%) patients in Group 1 and 14 (31.11%) patients in Group 2 (*P* = 0.8947). Vascular complications, such as venous thrombosis (involving the splenic or portal vein), were observed in 5 (20%) patients in Group 1 and 20 (43.5%) patients in Group 2 (*P* = 0.0479).

### Dependence between time of initiation of endotherapy and therapeutic outcomes

Negative correlations were observed between the time since the onset of ANP and the duration of active endoscopic drainage (R = −0.80, *P* = 0.0001); the time since the onset of ANP and the number of endoscopic interventions (R = −0.51, *P* = 0.003); and the time since the onset of ANP and the total duration of endoscopic treatment (R = −0.87, *P* = 0.0001).

## Discussion

The current guidelines on managing ANP recommend postponing endoscopic treatment of necrotic collection by at least 4 weeks since the disease onset^1,6–9,11,15^. In early phase of ANP, it is recommended to intensify conservative treatment with intravenous antibiotics, if necessary^16,17^, which can delay or even prevent surgical intervention^6^. However, some patients will still require interventional treatment for ANCs within first 4 weeks of ANP^11–15,26^. There are no data currently defining the role of endoscopic drainage in early phase of ANP. In the step-up approach, the recommended mode of interventional treatment is percutaneous drainage via transperitoneal or retroperitoneal access^10–12^. Present study demonstrated that endoscopic treatment for early necrotic collections is a successful method of treatment, which allows to limit or avoid additional access to the necrotic collections with other techniques.

The advantage of internal (endoscopic) over external (percutaneous) drainage is the lower risk of infection and no risk of pancreaticocutaneous fistula^10,12^. In contrast, percutaneous access allows for drainage irrespective of the location of the necrotic collection and is the least invasive technique. It can be performed at the bedside and does not require general anesthesia, which is important in critically ill patients at high risk of perioperative mortality and anesthesia-related complications^10–12^.

According to the step-up approach, percutaneous drainage of pancreatic necrosis is the first therapeutic step followed by extended access and video-assisted retroperitoneal debridement (VARD)^10–12^. In our facility, we try to avoid percutaneous drainage prior to endoscopic drainage. Early percutaneous drainage of pancreatic necrosis leads to evacuation of the liquefied necrotic contents with remnant solid contents within the collection, which prolongs treatment and requires earlier and more frequent debridement^26^. Furthermore, evacuation of the liquefied content with use of percutaneous drainage makes transmural endoscopic drainage difficult, if not impossible. Conversely, percutaneous drainage after previous endoscopic procedure with transmural drainage plays an important role in managing pancreatic necrosis by allowing for effective drainage and access to distant necrotic collections.

Herein, it is demonstrated that delayed endoscopic intervention for pancreatic necrosis until WOPN has developed leads to shorter duration of endoscopic treatment and fewer procedures, including endoscopic debridement and endoscopic necrosectomy. However, in some cases when surgical treatment is necessary in the early phase of acute pancreatitis, transmural endoscopic drainage of ANCs can be safely and successfully performed. Therefore, the paradigm that it is impossible to perform endoscopic treatment within the first four weeks of ANP was broken. Active endoscopic drainage of ANCs in early ANP is effective and allows to avoid surgery. When endoscopic drainage is ineffective, other methods of endoscopic treatment and more aggressive treatments can be considered according to the endoscopic step-up approach. If a patient still requires surgery, endoscopic drainage allows to delay surgery until WOPN develops and the patient’s condition improves.

Although present study demonstrated that endoscopic treatment of ANCs can be successful, endoscopic treatment of local complications of ANP in the early phase still pose clinical and therapeutic challenges for two main reasons. First, ANCs in early ANP are only partially walled or do not have a collection’s wall; therefore, endoscopic transmural gateways reach the retroperitoneal space where inflammation takes place. Second, ANCs mainly contains solid tissues bound to surrounding structures, which requires prolonged drainage and extra waiting time for liquefaction. These findings highlight the necessity for aggressive therapeutic treatments, more frequent endoscopic procedures, and prolonged endoscopic therapy compared with endoscopic treatment for WOPN.

According to Rana et al., endoscopic transmural drainage of necrotic collections containing > 40% of solid contents is reportedly associated with more complications and lower success rates^27^. In the present study, differences were unobserved in the efficacy and safety of the endoscopic treatment for ANCs and WOPN; therefore, adverse clinical outcomes were unconfirmed.

The choice of drainage technique in patients with necrotic collections should rely primarily on the experience of the treating medical center. Insertion of LAMS transmurally ensures wider transmural fistula, which makes the transmural drainage of necrotic collections more efficient. In our study during endotherapy we inserted LAMS in all cases of necrotic collections (both ANC and WOPN). The two most common and most serious groups of complications associated with the endoscopic treatment of necrotic collections with use of LAMS are gastrointestinal bleeding and perforations caused by leaking pancreaticogastric or pancreaticoduodenal anastomoses, which usually result from dislocation of the transmural stent. Both types of adverse events appeared in our study with similar rate in both groups. Despite the wall of ANC was not complete, large amount of adverse events connected with LAMS were not observed in group 1.

Two studies have attempted to explain the role of endoscopic treatment in ANCs in early ANP. Trikudanathan et al. demonstrated the therapeutic outcomes in a non-homogeneous group of 305 patients with ANP^28^. In their study, 193 (63.28%) patients required intervention, including within the first 4 weeks of ANP in 76 (39.38%) patients^28^. In those patients, either transmural endoscopic drainage and/or percutaneous drainage was performed^28^. Therefore, the results are complex to collate with the present study, considering endoscopic drainage was deemed the sole therapy for necrotic collections. However, minimally invasive strategies for WOPN were reported to be associated with a shorter hospital stay, lower mortality, and limited indications for emergency surgery^28^. Furthermore, patients who received endoscopic treatment for early ANP were described as requiring percutaneous drainage more frequently compared to patients with WOPN^28^, not observed in the present study.

In another study on endoscopic treatment in ANCs, Oblizajek et al. compared the outcomes of endoscopic treatment in 19 patients each with ANCs and WOPN^29^. They demonstrated similar efficacy and safety of endoscopic treatment for ANCs in the 3rd and 4th week of ANP compared with those of WOPN^29^. In present study, endoscopic drainage for ANCs was performed earlier, i.e., from the 2nd week of acute pancreatitis. Oblizajek et al.^29^ observed that the patients who underwent early endoscopic treatment for ANC required a longer stay in intensive care and longer endoscopic treatment than patients with delayed endoscopic intervention for WOPN. The authors did not report increased mortality, increased number of emergency surgeries, or increased number of patients requiring additional access to the necrosis^29^. Their results confirm our results of similar efficacy and safety of endoscopic treatment for ANCs in early ANP with longer endoscopic treatments, more aggressive treatments, and more frequent endoscopic procedures.

In our opinion, the size of necrotic collections does not reflect the full image of difficulties during the interventional treatment. Necrotic collections which do not extend outside the lesser sac are much easier to treat due to shorter endotherapy period and less aggressive approach. Opposite is the case of necrotic collections extending outside the lesser sac towards paracolic gutters or pelvic cavity.

Next issue worth considering is new type of transmural stents- electrocautery-tip LAMSs (EC-LAMSs)—which allows single-step EUS-guided drainage of pancreatic fluid collections^30,31^. This type of stent is a novel self-expanding metal stent^30,31^, which may be used in endotherapy of pancreatic necrosis without necessity to use other endoscopic tools during first endoscopic procedure (under performed cystogastrostomy or cystoduodenostomy)^30,31^. Currently two types of EC-LAMSs for various EUS-guided procedures are available: Hot-Spaxus (Taewoong Medical Co, Gimpo, Korea) and Hot-Axios (Boston Scientific, Marlborough, Mass, USA)^30,31^. In recently published article concerning EC-LAMSs, it has been proved, that novel EC-LAMS has high technical and clinical success rates for various interventional EUS indications, also for procedures of endoscopic transmural drainage of post-inflammatory pancreatic and peripancreatic fluid collections^31^. EC-LAMSs were not used in our study. Possibly, use of EC-LAMSs would improve efficacy and safety of early and late necrotic collections endotherapy.

The main limitations of present study are the lack of randomization and a relatively short duration of observation. Additionally, the investigation was conducted on a selected group of patients from a single medical center only. Nevertheless, all endoscopic interventions were performed by the same operator, which allowed for reliable comparisons of the results. Another limitation of our study is high rate of adverse events. Nevertheless, morbidity and mortality in case of endotherapy of necrotic collections in early phase of ANP is much smaller than morbidity and mortality in case of surgical treatment in early phase of ANP.

To conclude, the results of the present study justify postponing the endoscopic intervention in pancreatic necrosis, until liquefaction of necrotic content and the development of the WOPN. However, in urgent cases, endoscopic treatment can be effective also in early phase of ANP, which allows to avoid surgery and limits the need for additional methods of interventional treatment. Present study demonstrated the efficacy and safety of endoscopic treatment in early ANC; however, the approach was associated with a higher number of endoscopic interventions and prolonged drainage time than that in WOPN.
